# A Novel, Low-Cost, 3D-Printed Motorized Injector for Retinal Sheet Transplantation

**DOI:** 10.3390/bioengineering13020188

**Published:** 2026-02-06

**Authors:** Jerald Lim, Francis Ung, Samir Malhotra, Jacob C. Diaz, Austen Hamilton, Clare Chen, William C. Tang, Magdalene J. Seiler, Andrew W. Browne

**Affiliations:** 1 School of Medicine, University of California Irvine, Irvine, CA 92697, USA; jflim@hs.uci.edu; 2 Department of Ophthalmology and Visual Sciences, University of California Irvine, Irvine, CA 92697, USA; ungfp@uci.edu (F.U.); smalhot2@uci.edu (S.M.); jdiaz64@gwu.edu (J.C.D.); cchen448@uic.edu (C.C.); mseiler@uci.edu (M.J.S.); 3 Brunson Center for Translational Vision Research, University of California Irvine, Irvine, CA 92697, USA; hamil2am@mail.uc.edu; 4 Gavin Herbert Eye Institute, University of California Irvine, Irvine, CA 92697, USA; wctang@uci.edu; 5 Department of Anatomy and Neurobiology, University of California Irvine, Irvine, CA 92697, USA; 6 College of Medicine, University of Cincinnati, Cincinnati, OH 45267, USA; 7 College of Medicine, University of Illinois Chicago, Chicago, IL 60612, USA; 8 Department of Biomedical Engineering, University of California Irvine, Irvine, CA 92697, USA; 9 Department of Physical Medicine & Rehabilitation, University of California Irvine, Irvine, CA 92697, USA; 10 Sue and Bill Gross Stem Cell Research Center, University of California Irvine, Irvine, CA 92697, USA

**Keywords:** retina, transplant, instrument, organoid, 3D print

## Abstract

Retinal transplantation offers promise for restoring vision in advanced retinal degeneration. However, manual delivery of retinal sheets is often hindered by imprecise placement and collateral tissue damage resulting from instrument instability. We introduce a novel, 3D-printed, motorized retinal sheet injector designed to enhance placement accuracy and minimize tissue injury. The motorized injector features an Arduino-controlled foot pedal with three discrete actuator positions (“Min”, “Mid”, “Max”). When compared via frame-by-frame motion analysis, the motorized system reduced tip variance by approximately threefold over manual methods. In addition, in in vitro gelatin trials, the motorized injector achieved significantly higher placement accuracy versus the manual injector, which suffered from occasional complete misplacements. The novel motorized retinal sheet injector markedly improves stability and placement accuracy relative to manual methods, potentially reducing complications associated with subretinal delivery. Safer subretinal delivery can pave the way for innovative research and advanced treatment for retinal disease.

## 1. Introduction

Retinal degeneration in diseases like age-related macular degeneration (AMD) and retinitis pigmentosa results in irreversible loss of photoreceptors or retinal pigment epithelium (RPE), ultimately leading to blindness. Although early intervention is crucial to preserve vision, there are currently no broadly available therapies that halt progression during the early stages of these diseases. Therefore, advanced disease with cell death and vision loss remains common.

In advanced retinal degeneration, synthetic retinal stimulation offers a means to restore limited visual function. Bioelectronic prosthetics, such as the Argus II retinal prosthesis (Second Sight Medical Products, Inc., Sylmar, CA, USA), electrically stimulate surviving inner retinal neurons to evoke rudimentary visual perceptions [1]. Several groups have demonstrated the potential of devices that electrically stimulate the retina [2,3,4]. While bioelectronic prosthetics can elicit basic visual responses, they remain limited in their spatial resolution and non-physiological stimulation patterns.

Cell replacement therapy aims to restore retinal function by replacing lost cells through various strategies: transplantation of dissociated cells, cell sheets, or intact retinal tissue. For example, RPE transplantation has been performed by subretinal injection of dissociated human embryonic stem cell (hESC)-derived RPE in patients with AMD [5] and by transvitreal implantation of hESC-RPE on an ultrathin synthetic parylene substrate designed to mimic Bruch’s membrane [6], the latter approach reporting only mild, self-resolving adverse events. Other approaches include the transplantation of dissociated photoreceptors and the use of fetal retinal tissue or retinal organoid sheets, which have demonstrated functional integration in preclinical studies [7,8,9,10,11,12,13,14,15,16,17].

**Figure 1:**
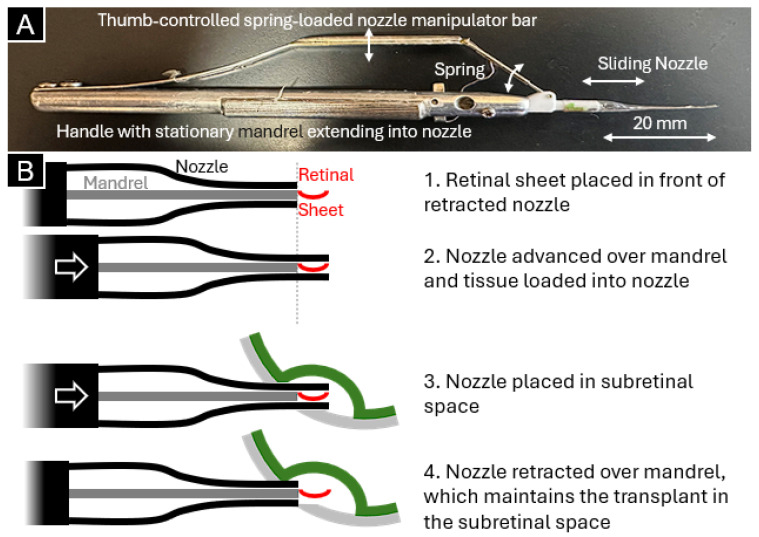
Manual injector (**A**,**B**) operating principles for transplanting sheets of retinal tissue into the subretinal space.

Despite these advances, achieving precise delivery of retinal tissue without collateral damage to the host tissue remains challenging. To improve placement precision, Aramant and Seiler developed a patented (Patent ID: 8057483) instrument that gently places the retinal sheet into the subretinal space, reducing the risk of increased intraocular pressure from excess fluid [8,9]. The first-generation retinal sheet transplantation instrument (Figure 1A) was designed with a pencil-like handle with a stationary, flat mandrel extending from the handle to the instrument’s tip. A polytetrafluoroethylene tip was manually fashioned from heat-shrink tubing over a mandrel mold and placed over the mandrel like a sock with an opening at the end. A thumb-controlled lever with a spring was used to manipulate the position of the nozzle over the fixed mandrel. Operation of the instrument (Figure 1B) involved placing a sheet of retinal tissue in front of the mandrel tip while the nozzle was moved from a retracted position to advance over the retinal sheet. The thumb-controlled manipulator was then locked into place with a clasp. To inject the retinal sheet into the subretinal space, the loaded nozzle was placed into the subretinal space, the thumb-controlled lever was unlocked by delicately squeezing the manipulator bar, and the nozzle was retracted over the retinal sheet by again delicately releasing all the spring tension applied to the manipulator bar. Meanwhile, the mandrel remained fixed in place to hold the retinal sheet in place. The process of manually unlocking and releasing the spring-loaded bar is nearly impossible to perform without small movements by the operator’s hand, and small movements on the handle result in larger movements at the instrument tip. This uncontrollable movement can affect transplant placement precision and overall success by causing damage to surrounding tissues.

Their method successfully facilitated the replacement of damaged photoreceptors with functional photoreceptor progenitors from fetal retinal sheets and hPSC-derived retinal organoids and led to some visual recovery in retinal degenerative models [8,9,10,11,12,18]. However, a significant number of transplants—especially transplants derived from retinal organoids—still resulted in rosette formation complications (i.e., photoreceptors arranged as spheres, with outer segments in their center). Additionally, improper positioning of the transplant within the vitreous by the user further contributed to this issue, emphasizing the need for an automated instrument capable of highly precise movements and accurate graft placement. To address these unmet needs, we developed a motorized injector designed to enhance placement accuracy while minimizing tissue damage. By integrating an Arduino interface with 3D-printed and off-the-shelf components, we created a high-precision instrument that remains cost-effective and highly accessible for researchers.

## 2. Materials and Methods

### 2.1. Design of Motorized Injector System

**Figure 2:**
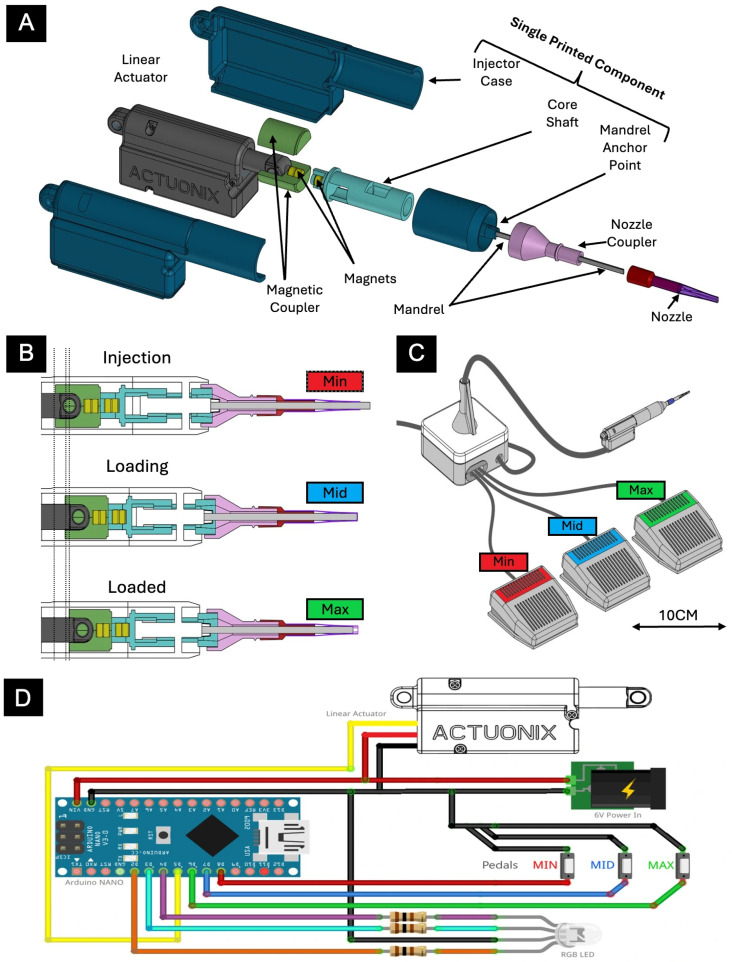
Design of motorized injector and system. (**A**) Exploded view of the actuation assembly. (**B**) Nozzle actuation mechanism. Small movements of the linear actuator, transmitted through the magnetic coupler, displace the nozzle coupler and nozzle over a stationary mandrel. (**C**) Schematic of fully assembled injector system. The assembled injector features three actuator petals controlled by an Arduino, achieving minimum, middle, and maximum nozzle positions. (**D**) Wiring diagram for Arduino microcontroller.

**Figure 3:**
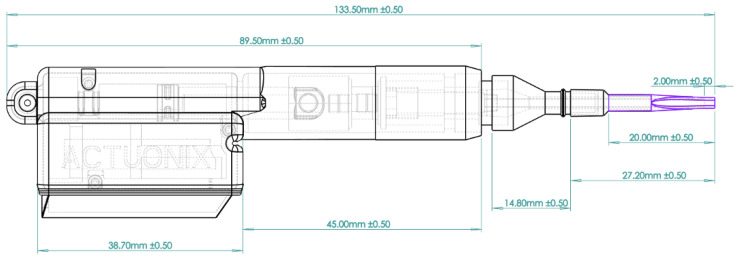
Dimensions of Injector. Assembly of the injector is shown in the figure above. Dimensions of the different parts of the injector are depicted.

The overall assembly and operation of the system are illustrated in Figure 2, whereas dimensions of the assembly are shown in Figure 3. The motorized injector was designed using a print-in-place approach that minimizes post-processing assembly.

The device integrates commercially available components with custom-machined metal, plastic, and 3D-printed parts to reduce costs while ensuring high performance. The primary components—including the injector case, core shaft, and mandrel actuator point—were fabricated with a Formlabs 3B (Formlabs, Somerville, MA, USA) printer using BioMed resin, which is biocompatible and autoclavable. An off-the-shelf linear actuator, typical of hobbyist applications, is magnetically coupled to the core shaft via a custom 3D-printed magnetic coupler, ensuring a secure connection while preventing over-retraction beyond the fully retracted state and damage to the system assembly.

The nozzle assembly is actuated through controlled, incremental displacements transmitted from the linear actuator to the core shaft. The nozzle coupler slides along a fixed stainless steel mandrel embedded within the mandrel anchor point. The nozzle is produced using a conventional ferrule technique, in which a heat-shrinkable fluorinated ethylene propylene (FEP) nozzle is loosely fitted over the mandrel and heated, yielding a conformal fit optimized for injectable organoids. In terms of the nozzle component, stainless steel was ultimately selected for its superior rigidity and biocompatibility compared to alternatives. Based on the manual Aramant and Seiler injector, our motorized injector was designed for 0.7 × 1.2 mm tissue. However, its modular design accommodates different nozzles for use of tissues of various sizes.

The linear actuator used is the PQ12-100-6-R (Actuonix Motion Devices Inc., Victoria, BC, Canada). Tested and operated at a 6 V input, the actuator reaches a maximum speed of 10 mm/s without load. Device safety is ensured through hardcoded software limits and physical travel constraints established by the housing neck.

Both the injector and electronics units are fully enclosed, with sealed I/O ports and wiring to facilitate sterilization and maintenance. Control of the injector is achieved through three actuator petals interfaced with an Arduino microcontroller, complemented by a foot pedal interface that provides three positional commands—labeled “Min,” “Mid,” and “Max”—to enable hands-free operation during surgical procedures. As we prioritized design of the handheld motorized injector, the three-positional pedal interface was selected as it was most practical for lab implementation. The system was tested and calibrated to ensure consistent nozzle advancement and retraction speeds.

**Figure 4:**
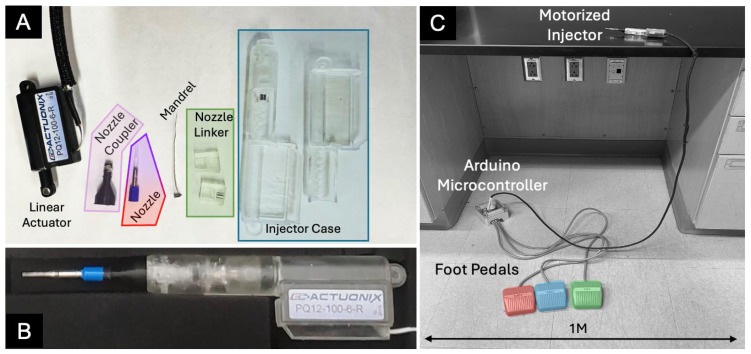
Photographs of motorized subretinal injector system with (**A**) disassembled components of the handheld motorized injector, (**B**) the assembled injector, and (**C**) the fully assembled system.

The manufactured components and assembled system are illustrated in Figure 4. Computer-aided design files for 3D-printed parts are available in the supplemental materials (Supplemental Materials: Surgical Injector Handheld Unit.step and Surgical Organoid Injector System.step).

### 2.2. Measurement of Injector Tip Movement

To quantify and compare the instrument nozzle tip movement for both the manual and motorized instruments, we recorded videos of the nozzle tip’s movement during operation. Four participants were tasked with operating both the manual and automatic injectors by fully extending and retracting the mandrel three times. Each of the participants was asked to stabilize their hand on a wrist rest throughout the trials while a video was recorded with an iPhone 14 Pro on a tripod (Apple Inc., Cupertino, CA, USA). Video frame-by-frame analysis was performed using Tracker, a free video analysis and modeling tool developed by Open Source Physics [19]. The position of the tip of the mandrel was plotted in a Cartesian plane. The variances in position were calculated and an F-test for equality of variances was performed to determine statistical significance. The average and maximum velocities were also calculated and compared between devices for each participant. Data were organized and summary statistics were calculated using Microsoft Excel (Version 2511, Microsoft Corporation, Redmond, WA, USA).

### 2.3. Accuracy of Retinal Sheet Placement

We evaluated, in vitro, the injector’s accuracy through placing a simulated sheet of retina on a target location. To do this, we used a surrogate system using Knox original unflavored gelatin. This model was chosen because it is a controlled, reproducible, low-cost preclinical test to evaluate the device before beginning in vivo trials. Incremental preclinical testing is the accepted path before animal and ultimately human use. We embedded pieces of 1 × 2 mm paper into this gelatin model to a depth of 20 mm beneath the gelatin surface. Smaller pieces of paper (0.7 × 1.2 mm) were then loaded into the manual or motorized injector nozzles and participants were tasked with placing and injecting the smaller piece of paper onto the larger piece of paper embedded in gelatin. All tasks were performed by participants using a dissection microscope (Leica MZ9.5) and repeated by each participant three times for each injector. The areas of overlap between the larger and smaller pieces of paper were quantified from photographs using ImageJ (Version 1.54g). The percentage of area overlap was used to represent placement accuracy and cumulative results were plotted.

## 3. Results

**Figure 5:**
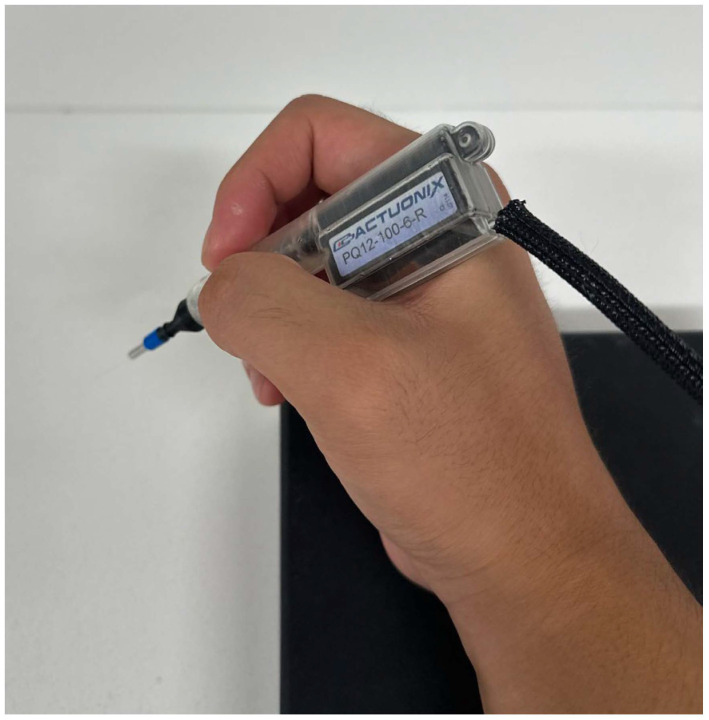
Motorized Injector in Hand. Photograph of motorized subretinal injector system in hand.

The manufactured components and fully assembled motorized injector and operational system are shown in Figure 3. The motorized injector in hand is shown in Figure 5.

### 3.1. Measurement of Injector Tip Movement

**Figure 6:**
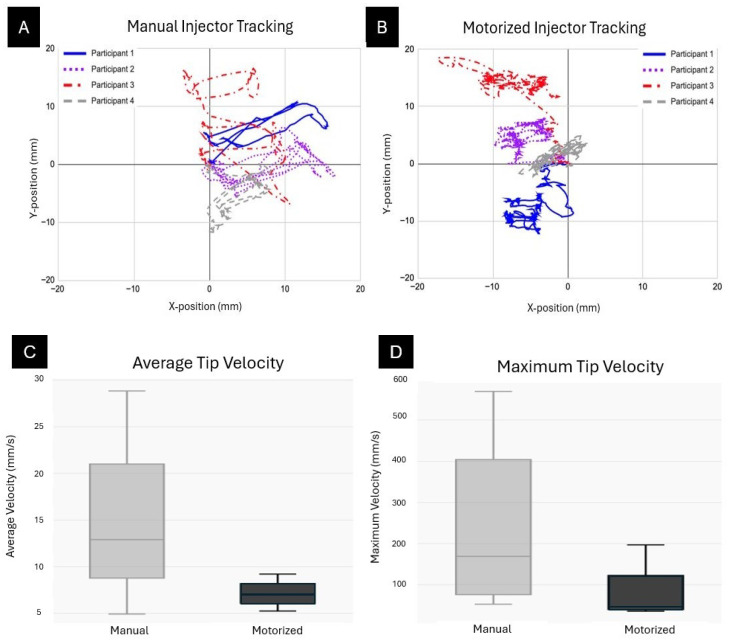
Injector Tip Tracking Trials. The tips of the injectors were tracked and the positions plotted on a cartesian plane. (**A**) represents the manual injector and (**B**) represents the motorized injector. The pathways of each participant are plotted on the same graph. Average tip velocity (**C**) and maximum tip velocity (**D**) for each participant are represented on a box plot and separated by type of injector.

Figure 6 illustrates the tracked tip trajectories and velocity distributions for both manual and motorized instrument types. Panels A and B display the Cartesian plots of tip positions for the manual and motorized injectors, respectively, with all participant pathways overlaid. Notably, manual trials exhibited a roughly threefold greater dispersion in tip positions compared to motorized trials (all F-tests, *p* < 10^−11^). Quantification of average and maximum tip velocities (Figure 6C,D) demonstrated lower velocity variability for the motorized system, while tip velocity of the manual system showed about twice that of the motorized one. However, differences in tip velocities were not statistically significant (one-tailed *t*-tests, *p* > 0.1).

### 3.2. Accuracy of Retinal Sheet Placement

**Figure 7:**
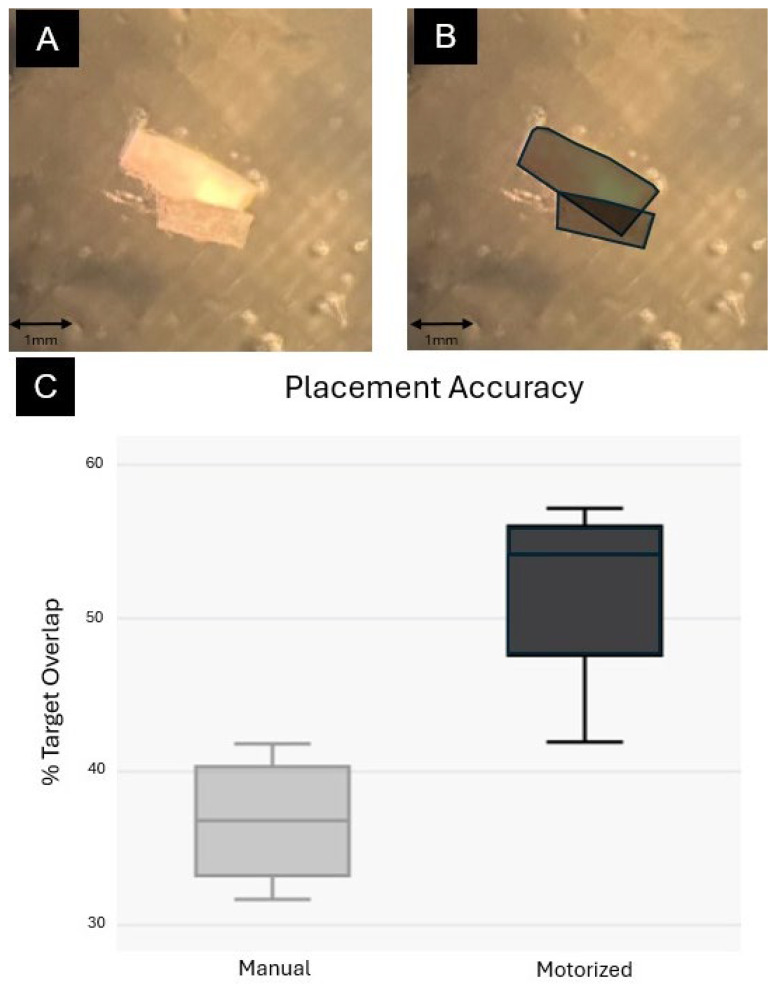
Injector Accuracy Trials. Each participant was instructed to place an injectable piece of paper over a landing strip. The % target overlap was then calculated by calculating the area of the paper overlapping with the landing strip with the total area of the injectable. (**A**) A single trial of the injectable over the target landing strip. (**B**) Dark coloring is applied to highlight the overlapping areas—the area of overlap was calculated and then divided by the total area of the injectable to achieve % target overlap. (**C**) Percent target overlap for each trial represented in a box plot for manual vs. automated injector.

Figure 7 summarizes placement accuracy across all four participants. A representative trial and the method for calculating percent target overlap by dividing the overlapping area of the injectable by its total area is illustrated in Figure 7A,B. Overall, the motorized injector yielded significantly higher accuracy than the manual injector, with average accuracies of approximately 52% (95% confidence interval: 44.9–58.7%) and 37% (95% confidence interval: 19.42–54.11%), respectively (one-way ANOVA, F = 13.75, *p* = 0.00999, n = 8, η^2^ = 0.696, Cohen’s *d* = 2.62) (Figure 7C). Notably, some manual trials resulted in complete misplacement (0% overlap). During all injection trials, the pieces of paper remained stable during manipulation and leakage did not occur. Hydrostatic forces help to retain samples inside the injector.

## 4. Discussion

Normal human physiologic tremor and the limitations of hand–eye coordination can cause complications during medical procedures, especially in microsurgery. Implementation of novel devices to mitigate tremor has helped with this problem. Taylor et al. (1999) pioneered tremor-reduction technology by creating a robotic arm that steadies surgical instruments while the surgeon retains control [20]. Likewise, Intuitive Surgical’s da Vinci^®^ Surgical System—also introduced in 1999—employs a master-slave robotic interface that filters out the surgeon’s hand tremor during minimally invasive procedures [21]. This device, widely used in minimally invasive surgery, gives the surgeon remote control of robotic arms via a console. The machine is then able to filter any tremors and prevent translation from the operator to the robotic arms. Integration of technology into surgery can reduce operator variability and result in improved surgical outcomes [22].

Earlier investigations of physiologic tremor in vitreoretinal microsurgery found a tremor amplitude of ~100 um at the tip of the instrument [23,24]. Thus, the motor skills required for working in the subretinal space approach the limit of human physiological tremor. Mitigating physiologic tremor is critical in microsurgery, particularly for subretinal injections. Manual delivery—even by expert surgeons—carries a high risk of complications. Precise placement is challenging: slight misalignment can perforate Bruch’s membrane and enter the choroid, while the retina’s limited elasticity means minor tip movements can enlarge retinotomies. Additional hazards include inaccurate dosing, inadvertent injection into the vitreous, and retinal detachment. For example, previous studies have shown that in human trials, retinal sheet transplantation has resulted in complications such as epiretinal membrane formation, subretinal hemorrhage, and macular holes. Some patients have even presented with misplaced grafts relative to normal RPE, resulting in fibrosis of the transplanted tissue [25,26]. While tremor cannot be fully eliminated, the device significantly reduces unnecessary movements and hopefully the incidence of trauma-induced complications. In addition, increased baseline accuracy will reduce the incidence of misplacement complications.

Previously, in subretinal drug delivery, surgeons attempted to mitigate these problems by holding the injector cannula and having an assistant depress the syringe plunger. However, this method introduces a second operator and therefore is also prone to inconsistency and operator induced complications. To reduce adverse effects when delivering drugs into the subretinal space, MedOne developed the MicroDose injector, which connected a subretinal cannula to a vitrectomy machine to allow actuation via foot pedals. Usage of this device resulted in an increase in precision, accuracy, and reliability as the surgeon’s hands are allowed to concentrate on cannula placement [21]. Some have also developed and improved their own subretinal transplantation instruments. For example, Fernandes et al. developed a prototype and improved injector. Their previous-generation injector was a 15 gauge injector, and they decreased the size of the needle to 17 gauge to decrease damage to the implant and the retina. Compared to their previous model, they noted a decrease in retinal bleeding and decreased surgical time. In addition, because their new instrument was able to unfold and position the substrate with a single instrument, the procedure became easier, more reliable, and reproducible. Although their next-generation injector was undoubtedly an improvement upon their older injector, given the instrument’s manual operation, it is still somewhat at risk for traumatic complications related to human tremor [27].

The patented Seiler and Aramant retinal sheet injector is also prone to these complications because of its manual operation. Therefore, we developed a 3D printed, motorized injector to mitigate operator-induced variability and increase the success rate of retinal sheet transplantation. The motorized subretinal sheet injector replaces manual plunger motion with an Arduino-controlled linear actuator operated by foot pedals. This configuration suppresses surgeon tremor, delivering smoother nozzle movements while retaining biocompatible, sterilizable construction. In bench-top stability tests with four operators, tip-position variability fell significantly compared with the manual device. Mean tip velocities were similar, but the motorized injector trended slower—an effect that may further limit graft and retinal trauma.

In simulated transplantation tasks, the motorized system correctly positioned retinal sheets in 52% of trials versus 37% for the manual injector; complete misplacements occurred only with the manual tool. These findings point to greater placement precision and fewer procedure-related complications with motorized actuation.

The injector’s housing and key mechanical parts can be fabricated with standard 3D printers and assembled from off-the-shelf Arduino, pedal-switch, and linear-actuator components. This low-cost, open-architecture design allows other laboratories to replicate or adapt the device rapidly—facilitating broader adoption of precision subretinal delivery for gene, cell, or drug payloads without reliance on proprietary hardware.

Despite these promising results, there are several limitations. The study was conducted with only four participants, and the in vitro model may not fully replicate in vivo conditions. Additionally, operator variability and learning effects were not comprehensively addressed, and the clinical relevance of the measured parameters requires further validation in animal models. While the device is currently designed for rodent transplantation, its modular construction with swappable parts allows for future scaling to human application.

## 5. Conclusions

Our findings demonstrate that the motorized injector significantly improves placement accuracy and operational stability compared to the manual injector. By reducing undesired hand tremor effects and enhancing control, this system shows promise for improving the outcomes of subretinal sheet transplantation. Future work should focus on validating these results in vivo. Furthermore, subsequent iterations of our device may incorporate robotic platforms or artificial intelligence models to assist in positioning, maximizing surgical accuracy and precision.

## 6. Patents

Patent Pending: UC-2025-837-1 “Motorized Retinal Transplant Delivery Device and Method of Use”; inventors: Andrew W. Browne, M.J. Seiler, W.C. Tang, Francis P. Ung.

US Patent # 8,057,483 (issued November 2011) “Subretinal Implantation Instrument”; inventors: R. Aramant, M. Seiler.

## Data Availability

The original contributions presented in this study are included in the article/Supplementary Material. Further inquiries can be directed to the corresponding author.

## Supplementary Materials

The following supporting information can be downloaded at: https://www.mdpi.com/article/10.3390/bioengineering13020188/s1.

## Abbreviations

The following abbreviations are used in this manuscript:

AMD	Age-related macular degeneration
RPE	Retinal pigmented epithelium
hESC	Human embryonic stem cell
